# Monoclonal Antibodies Targeting CGRP: From Clinical Studies to Real-World Evidence—What Do We Know So Far?

**DOI:** 10.3390/ph14070700

**Published:** 2021-07-20

**Authors:** Theodoros Mavridis, Christina I. Deligianni, Georgios Karagiorgis, Ariadne Daponte, Marianthi Breza, Dimos D. Mitsikostas

**Affiliations:** 1 1st Department of Neurology, Eginition Hospital, Medical School, National and Kapodistrian University of Athens, 11528 Athens, Greece; ariadne__89@hotmail.com (A.D.); marianthibr@med.uoa.gr (M.B.); dmitsikostas@med.uoa.gr (D.D.M.); 2 Neurology Department, Athens Naval Hospital, 11521 Athens, Greece; cdchristina@gmail.com; 3 Neurology Department, 401 Military General Hospital, 11525 Athens, Greece; giorgoskarmed@hotmail.com

**Keywords:** calcitonin gene-related peptide, CGRP, erenumab, fremanezumab, galcanezumab, eptinezumab

## Abstract

Now more than ever is the time of monoclonal antibody use in neurology. In headaches, disease-specific and mechanism-based treatments existed only for symptomatic management of migraines (i.e., triptans), while the standard prophylactic anti-migraine treatments consist of non-specific and repurposed drugs that share limited safety profiles and high risk for interactions with other medications, resulting in rundown adherence rates. Recent advances in headache science have increased our understanding of the role of calcitonin gene relate peptide (CGRP) and pituitary adenylate cyclase-activating polypeptide (PACAP) pathways in cephalic pain neurotransmission and peripheral or central sensitization, leading to the development of monoclonal antibodies (mAbs) or small molecules targeting these neuropeptides or their receptors. Large scale randomized clinical trials confirmed that inhibition of the CGRP system attenuates migraine, while the PACAP mediated nociception is still under scientific and clinical investigation. In this review, we provide the latest clinical evidence for the use of anti-CGRP in migraine prevention with emphasis on efficacy and safety outcomes from Phase III and real-world studies.

## 1. Introduction

Migraine is a common brain disease, classified as the second most debilitating condition and has the third highest prevalence of all medical conditions [1]. The last decade heralded a new era in migraine therapeutics. Recent advances in the field of migraine research have resulted in newly available treatment options. Among them are the anti-calcitonin gene-related peptide-receptor (anti-CGRP/R) monoclonal antibodies (mAbs). The four available anti-CGRP/R mAbs were the only disease-specific preventive agents that have the potential to change the migraine therapeutic background until now. Three of these macromolecules target the calcitonin gene-related peptide (CGRP) ligand (fremanezumab, galcanezumab, and eptinezumab), while a fourth (erenumab) targets the CGRP receptor [2,3,4]. The CGRP pathway plays a major not only in migraines, but in cluster headaches, post-traumatic headaches, fiblomyalgia and other pain conditions. Here, we present all current knowledge on anti-CGRP mAb clinical testing.

## 2. Erenumab

Erenumab or AMG 344 is the first anti-CGRP mAb which targets the CGRP receptor. The recommended dose is 70 mg or 140 mg every 4 weeks as a single autoinjection administered subcutaneously (sc). It is a fully human IgG2 monoclonal antibody and a potent, selective, and full competitive inhibitor of the CGRP receptor [5,6].

### 2.1. Clinical Studies

From Phase I studies [5,7,8,9], assessing the safety, tolerability, pharmacokinetics (PK), and pharmacodynamics (PD) of erenumab, it was shown that erenumab is a safe and well tolerable drug with linear PK for doses above from 70 mg, typical IgG2 mAb behavior, and a highly potent CGRP receptor inhibitor profile. At 70 mg, the estimated elimination half-life of erenumab is 21 days, supporting monthly subcutaneous dosing and thus improving patient compliance [5,8,10]. In another Phase I study, concomitant administration of erenumab and sumatriptan (sc) in healthy subjects had no negative effect on resting blood pressure and did not alter the PK of sumatriptan or raise any safety issues [7]. Considering that migraines mostly affect childbearing women, the potential interaction of concomitant use of erenumab and estrogen/progestin combination oral contraceptive was investigated. The results showed no effect in the PK of the contraceptives and their efficacy [9].

Considering that CGRP is a potent vasodilator, treatment with anti-CGRP or anti-CGRP receptor mAbs raises the question of potent cardiovascular risk [11]. In a Phase II double-blind placebo-controlled trial (DBTP) the effect of erenumab in patients with stable angina during treadmill exercise was assessed [12]. Results were encouraging, showing that inhibition of the CGRP receptor neither affected exercise tolerance nor worsened myocardial ischemia.

Furthermore, three Phase III and two Phase II randomized clinical trials (RCTs) for prevention of episodic migraine (EM) [13,14,15,16,17] and one Phase IIb RCT for the prevention of chronic migraine (CM) were conducted [18], lasting from 12 weeks up to 6 months. Treatment with erenumab proved to be effective, safe and well tolerated as a preventive medication. Regarding effectiveness, a pooled analysis including data from five studies with 70 mg and/or 140 mg erenumab showed an increased rate of 50% reduction and a decrease in the mean monthly migraine days (MMDs) (MD—1.32; *p* < 0.00001; I2 = 100%) and in migraine-specific medication days (MD—1.41; *p* < 00001; I2 = 100%) from baseline in the erenumab group compared with the placebo group [19]. An additional benefit from erenumab was observed in functional outcomes even in patients with EM and previous 2–4 preventive treatment failures [20].

Data from up to five years of the extension to open-label phase of three Phase III RCTs (OLTP) [14,17,18] are available [21,22,23,24,25], confirming the consistency of sustained efficacy in prevention of EM and CM and safety profile, while highlighting the stability of the improvements in functional outcomes and migraine-related quality of life scoring for at least 5 years [22].

Mean (S.E.) change in MMDs from baseline of 8.7 (0.2) days was −5.3 (0.3) days with a reduction of 62.3% at year 5. At the end of 5 years [22] of extension of the DBTP for EM, mean change in monthly migraine-specific medication days was −4.4 (0.3) days, among patients using acute migraine-specific medication at baseline (6.3 (2.8) treatment days). Meanwhile, one year data from the OLTP [25] showed that the reduction rates of 50%, 75% and 100% from the DBTP baseline in MMD at week 52 of OLTP were 59.0%, 33.2% and 8.9%, respectively, for the combined dose group. Discontinuation rates in all studies were low either due to non-effectiveness or AES.

### 2.2. Real-World Evidence

So far, published data from seven observational studies have been published [26,27,28,29,30,31,32]. All studies evaluated the efficacy and safety of erenumab [26,28,29,30,31,32] in patients with CM and EM [26,30], with at least 8 weeks and up to 9 months of treatment, who had more than three previous medication failures. In all studies, erenumab proved to be an efficacious and well tolerated medication with low discontinuation rates. Response rates were higher after month three of administration, and combination of erenumab with other preventive treatments (oral or OnabotulinumtoxinA) seemed to be more effective in refractory patients [29].

Higher doses of erenumab might have given a substantial contribution to patients with a higher number of baseline MMDs or with medication overuse [28,30].

Additionally, an observational study including refractory CM patients measured spinal sensitization using the temporal summation threshold (TST) of the nociceptive withdrawal reflex and assessed the inflammatory biomarker profile by measuring micro-RNA subtypes in blood samples. The authors concluded that different responses to treatment may result from different neurophysiological and biomolecular behaviors between responders and non-responders [27].

### 2.3. Adverse Events

Generally, AEs were mild to moderate and transient, mainly experienced in the first months of the treatment and decreased overtime [28]. The exposure-adjusted patient incidence rate of AEs was estimated to be 123.0/100 patient-years while the exposure-adjusted incidence rate of SAEs was 3.8/100 patient-years [22]. SAEs were mostly isolated cases with clearly related patterns [21]. No difference was detected between doses of 70 mg and 140 mg (risk of any AE; RR—1.0) [19]. The most common AEs included nasopharyngitis/viral upper respiratory tract infection, upper respiratory tract infection, influenza, and injection site pain reaction in DBTP and OLTP studies [10]. Nevertheless, in real-world evidence (RWE) studies, it was found that constipation was the most frequent AE [28,30]. Long-term treatment with erenumab does not affect liver function. So far, no association between erenumab and vascular events is proven according to a pooled analysis [33]. Although from DBPT and OLTP studies no meaningful changes in blood pressure were noticed, data from RWE and a retrospective analysis of postmarketing case reports may show a potent association between erenumab treatment and hypertension [28,34], indicating that although not clear yet, special attention should be paid.

### 2.4. Beyond Migraine and Future

Data from a 12-week open-label study investigating the reduction in monthly headache days (moderate to severe) in adults with persistent post-traumatic headaches (PTHs) with administration of 140 mg erenumab every 4 weeks showed a lower frequency of monthly headache days by the end of the study [35]. A Phase I DBPT has been completed evaluating the efficacy, safety, tolerability, and PK of erenumab in women with hot flashes associated with menopause; nevertheless, the results are not published. Finally, an OLTP is ongoing studying the efficacy and tolerability of erenumab in the prevention of persistent redness and flushing of patients with rosacea (clinicaltrials.gov, assessed on 24 May 2021).

## 3. Galcanezumab

Galcanezumab or LY2951742 is a monoclonal antibody that targets and binds CGRP, therefore inhibiting its physiological activity. It is an IgG4, a 90% fully humanized antibody to CGRP [36,37,38].

It has linear pharmacokinetics and steady-state concentrations that are achieved by the first month, when a loading dose of 240 mg followed by a 120 mg maintenance dose is administered. It is unique among the four monoclonal anti-CGRP antibodies, since it is the only one that has proven efficacy as a preventive treatment of cluster headaches [39].

### 3.1. Clinical Studies

In early clinical development, Vermeersch et al. assessed the target engagement and dose selection of galcanezumab, which correctly predicted inhibition of capsaicin induced dermal blood flow in humans starting at a single subcutaneous 5 mg dose [40]. Another Phase I study evaluated the safety and tolerability of single and multiple doses of galcanezumab in humans. Galcanezumab was well tolerated with linear pharmacokinetics. The study did not find any dose-related adverse effect or signs of cardiovascular adverse effects or hepatotoxicity [41].

A Phase II study for EM prevention showed the efficacy of galcanezumab and its superiority over placebo (MMD: −4.2 vs. −3, *p* = 0.003). There was also a statistically significant increase in the response rate compared to the placebo (odds ratio: 2.88 90% CI: 1.78–4.69). [42]. In another Phase IIb study, Skljarevsk et al. evaluated the effect of different doses of galcanezumab in patients with EM. They found that 120 mg sc galcanezumab significantly reduced migraine headache days compared to the placebo (99.6% posterior probability: −4.8 days; 90% BCI, −5.4 to −4.2 days vs. 95% superiority threshold (Bayesian analysis) −3.7 days; 90% BCI, −4.1 to −3.2 days) [43,44].

Similar results were reported in a Phase II RCT evaluating the efficacy of galcanezumab in Japanese patients with EM. Both the 120 mg and 240 mg galcanezumab showed statistically significant (*p* < 0.001) improvements in the overall mean change in monthly migraine headache days. Additionally, patients in both galcanezumab groups reported statistically significant response rates and fewer acute-medication days compared with the placebo group [45].

EVOLVE-1 and EVOLVE-2, two phase III RCTs, showed the efficacy of galcanezumab in patients with EM [46,47,48]. Both studies revealed that patients in both galcanezumab groups reported less migraine headache days compared with the placebo group [46,47,48]. Moreover, in the EVOLVE-1 study, 50%, 75%, and 100% reductions in migraine days were reported in 62%, 39%, and 16% of patients in the 120 mg group and in 61%, 39%, and 15% of patients in the 240 mg group, which are both statistically significant compared to the placebo group (*p* < 0.001). Interestingly after cessation of treatment and during the 4-month post-treatment follow-up, migraine headache days remained significantly lower than the placebo group in both galcanezumab groups (*p* < 0.05) [46,47,48]. Another Phase III clinical trial, which studied the safety of galcanezumab in patients with EM and CM, had encouraging results, with no statistically significant differences in laboratory values, vital signs and electrocardiogram recordings between groups. Again, patients in both groups reported fewer migraine headache days compared to baseline and used less acute medication for migraine attacks (*p* < 0.01) [49].

Consistency of results was also present in CONQUER and REGAIN studies, Phase III double-blind studies, which assessed galcanezumab in treatment-resistant patients with migraines (episodic and chronic). At the primary endpoint (overall mean change in the number of migraine headache days per month from baseline), both doses of galcanezumab were superior to placebo group [50,51].

### 3.2. Real-World Evidence

TRIUMPH, a long-term, real-world evidence study of galcanezumab was initiated in December 2019 on behalf of Eli Lilly and Company. The purpose of this study is to assess the real-world effectiveness of galcanezumab in contrast to other preventive medications for migraine.

### 3.3. Adverse Events

The adverse effects of galcanezumab were mild and self-limited. There was also a consistency among all studies. Most common AEs are injection site reactions, nasopharyngitis, sinusitis, back, neck and extremity pain and urinary tract infections [42,44,45,49,51].

### 3.4. Beyond Migraine and Future

In a randomized, double-blind Phase III trial, Goadsby et al. evaluated the efficacy of galcanezumab in patients with episodic cluster headaches. Patients in the galcanezumab group experienced a significant reduction in the weekly frequency of cluster headache attacks (mean reduction across weeks 1 to 3: −8.7 attacks) compared to placebo (mean reduction: −5.2 attacks, *p* = 0.04). No serious adverse effects were observed, and mild-to moderate adverse effects had a higher frequency in the galcanezumab group (43% vs. 33%), but there was no significant difference in the discontinuation rate due to adverse effects between the two groups [39]. In another Phase III randomized, placebo-controlled study, Dodick et al., assessed the efficacy and safety of galcanezumab in adults with chronic cluster headaches, but unfortunately the study did not meet either its primary or secondary endpoints [52].

An ongoing randomized, double-blind, placebo-controlled Phase III study of galcanezumab investigates its role in 6-year-old to 17-year-old patients with episodic migraines [53]. Another ongoing Phase I clinical study focuses on assessing the potential for a pharmacokinetic interaction. The main purpose of this study is to provide safety and tolerability information when ubrogepant and erenumab or ubrogepant and galcanezumab are co-administered [54].

In another single-group open-labelled study, patients suffering from chronic migraine will be enrolled, and the main goal of this trial is the discovery of the primary pharmacodynamic target and whether this novel and recently approved class of migraine prophylactic drugs act inside or outside the brain and if so, where [55]. More open-label and real-world evidence data are pending. Specifically, there is currently an ongoing study that aims to evaluate long-term safety and tolerability of galcanezumab. Galcanezumab is administered up to once monthly in patients suffering from episodic or chronic cluster headaches who have completed the two previous clinical trials on cluster headache. The duration of the study will be approximately 4 years [56].

## 4. Fremanezumab

Fremanezumab or TEV48125 is a fully human immunoglobulin G2 (IgG2) delta a/kappa antibody (mAb) that selectively targets both isoforms, α and β, of CGRP. Preclinical studies using dose-dependent inhibition of intracellular cAMP release, induced by CGRP, showed that fremanezumab interferes with the ability of CGRP to bind and signal through its receptors [57].

### 4.1. Clinical Studies

Five Phase I studies evaluated the safety and tolerability of fremanezumab and revealed a consistently good safety profile, using doses ranging from 0.2 mg to 2000 mg [58]. The cardiovascular safety of fremanezumab was also reported in an older female population at the high dose of 2000 mg [4].

A Phase II study in 2015 showed the efficacy of two different doses of fremanezumab in patients with high-frequency EM [59]. A post hoc analysis showed a 50% reduction in the MMD in 28% of the placebo group compared with 53% in the 225 mg group (*p* = 0.0005) and 59% of the 675 mg group (*p* < 0.0001).

The Phase II study, assessing the efficacy of fremanezumab in patients with CM, had similar results, showing improvements in both primary and secondary endpoint measures relative to the placebo group [60]. Subcutaneous TEV-48125 significantly decreased the number of headache hours, the number of moderate to severe headache days and the acute symptomatic medication needed for migraine attacks within the first month. Post hoc analyses were conducted based on pooled data from these two Phase II studies [61,62]. Fremanezumab was found to be safe as an add-on preventive therapy and compatible with all major classes of migraine preventive therapies.

The HALO study was a Phase III, randomized, double-blind, parallel group study comparing fremanezumab to placebo in EM patients with two or less previous treatment failures. Subcutaneous fremanezumab resulted in a statistically significant day reduction in the MMD, an increase in 50% reduction in the mean number of monthly migraine days and a reduction in migraine-specific acute medication over a 12-week period compared with the placebo group (*p*-value < 0.0001). Additionally, fremanezumab reduced migraine-associated, sometimes debilitating, symptoms such as nausea or vomiting, photophobia, and phonophobia compared with the placebo group [63].

The efficacy of fremanezumab, as a preventive therapy in CM, was assessed in a Phase III RCT study [64]. Fremanezumab resulted in a reduction of at least 50%, in the average number of headache days per month in 38%, 41% and 18% in the fremanezumab-quarterly, fremanezumab-monthly and placebo groups, respectively (*p* < 0.001 for both comparisons with placebo).

The FOCUS study was a randomized, double-blind, Phase IIIb trial that showed the drug’s efficacy in difficult to treat, refractory cases with EM and CM [65].

The first study investigating long-term efficacy and safety of fremanezumab, in both CM and EM, revealed the sustainability of the drug’s effectiveness for at least one more year [66].

### 4.2. Adverse Events

In early clinical studies, treatment-related adverse events occurred in 21.2% of subjects receiving fremanezumab (no association pattern regarding the dosage), compared to those receiving the placebo (17.7%) [59,63]. Most common treatment-emerging AEs were injection site pain, headache, nasopharyngitis, gastroenteritis, and back pain. Liver abnormalities were encountered slightly more often (1%) in the treatment groups compared to placebo (<1%), which can be attributed to the use of non-streroidal anti-inflammatory drugs [63]. The frequency of adverse events was similar to that of the placebo group and less than 1% of patients in the fremanezumab group had adverse events leading to discontinuation [65]. Fremanezumab was not associated with any clinically relevant patterns of change in vital signs (systolic and diastolic blood pressure, temperature and heart rate). Additionally, the studies did not reveal any changes in ECG parameters or laboratory findings. Moreover, similar findings resulted from Phase I studies comparing safety issues between healthy Caucasian and Japanese subjects [67].

### 4.3. Beyond Migraine and Future

Fremanezumab was also investigated for other headache conditions. A Phase III clinical trial on both episodic and chronic cluster headaches was unfortunately discontinued following a prespecified futility analysis that revealed that the primary endpoint (i.e., mean change from baseline in the weekly average number of cluster headache attacks) during the 4-week treatment period, was unlikely to be met [68]. Additionally, two Phase IV studies have been initiated, the first enrolling patients with migraine and major depressive disorder [69] and the second with migraine and CADASIL [70,71]. The effect of fremanezumab has been suggested for other pain conditions as well. There are ongoing clinical trials on fibromyalgia [72], Post-traumatic Headache (PTH) [73] and interstitial cystitis-bladder pain syndrome [74]. Additionally, a Phase IV, open-label study has been designed aiming to evaluate the efficacy of fremanezumab on interictal migraine-related burden [75]. Moreover, studies using self-administration of subcutaneous fremanezumab [76] and studies on younger age groups 6 years old to 17 years old with EM or CM are currently being coordinated [77,78].

## 5. Eptinezumab

Eptinezumab or ALD403 is a humanized anti-calcitonin gene-related peptide IgG1 monoclonal antibody that binds to calcitonin gene-related peptide (CGRP). It selectively binds α- and β-forms of human CGRP ligand to prevent activation of the CGRP receptor and blocks its binding to the receptor for the prevention of migraine [79]. Eptinezumab is delivered by intravenous (IV) administration (100 mg) every 3 months and has a plasma half-life after an intravenous infusion of 100 mg of 31 days.

### 5.1. Clinical Studies

Eptinezumab has been evaluated in five clinical trials (four randomized double-blind, placebo-controlled, parallel-group studies and one open-label, long-term safety study) conducted in patients with migraine, which demonstrated the efficacy and safety of eptinezumab. The primary endpoint was the change from baseline in mean MMD over months 1–3. It seems to be well tolerated so far, with 1.7% of infrequent serious AEs (placebo 1.4%). Only 1.9% of patients treated in the PROMISE-1 and PROMISE-2 trials were discontinued due to adverse events (AEs) [80].

PROMISE-1 clinical trial that was completed was a Phase III, randomized, double-blind, placebo-controlled, study evaluating intravenous 30, 100, and 300 mg eptinezumab or placebo for the prevention of episodic migraine [81]. In PROMISE-1, a total of 665 patients suffering from episodic migraine were randomized to receive placebo, 100 mg eptinezumab, or 300 mg eptinezumab every 3 months for a duration of 12 months. The mean frequency at baseline was ~8.6 migraine days/month. The study concluded that the mean reduction in days from baseline to placebo was −3.9 migraine days for 100 mg, −4.3 days for 300 mg and −3.2 days for placebo [82].

In PROMISE-2, which was a clinical trial that recruited a total of 1072 patients with chronic migraine. Migraine sufferers were randomized to receive placebo, 100 mg eptinezumab, or 300 mg eptinezumab every 3 months for 6 months [83]. The mean frequency at baseline was approximately 16.1 migraine days/month. The study results demonstrated that the mean reduction in migraine days from baseline to placebo was −7.7 days for 100 mg, −8.2 days for 300 mg, and −5.6 days for placebo [84,85].

In PREVAIL, an open-label, Phase III clinical trial (NCT02985398) evaluating safety, 300 mg eptinezumab was administered every 3 months in a total of 128 patients with chronic migraine (a primary treatment phase, four infusions 12 weeks apart and a secondary treatment phase, ≤4 infusions 12 weeks apart) [86]. Reductions in HIT-6, migraine-related burden on work, home and social functioning (Migraine Disability Assessment) and better HR-QOL scores were reported (SF-36 PCS, MCS scores). Benefits were first observed at week 12 and sustained through to week 52 of treatment. The study enrolled adult patients with migraine ≤50 years of age and a history of chronic migraine for more than a year [87].

Overall, results from clinical trials were promising, concluding a statistically significant and clinically meaningful migraine preventive effect of eptinezumab in patients with episodic migraine over weeks 1–12 following the first IV administration. Safety and tolerability were compared to placebo. In PROMISE-1 and PROMISE-2, only 1.9% of patients discontinued treatment due to adverse reactions.

### 5.2. Adverse Events

Most adverse events were transient and severity was reported as mild to moderate. Patients treated with eptinezumab or placebo, presented no clinically significant differences in vital signs, 12-lead ECGs, or laboratory safety data. Likewise, the most commonly reported AEs for eptinezumab, at an incidence of ≥2%, were hypersensitivity reactions (hypersensitivity, urticaria, flushing/hot flush, rash, and pruritus), nasopharyngitis (6.3%), upper respiratory infection (4%), nausea (3.4%), arthralgia (2.3%), urinary tract infection (3.1%), dizziness (2.6%), fatigue (2%) and anxiety (2%) [84,85,88].

### 5.3. Beyond Migraine and Future

RELIEF, a randomized, double-blind, placebo-controlled trial, is recruiting migraine sufferers to evaluate the efficacy of a single dose of eptinezumab 100 mg in patients experiencing acute migraine attacks [89].

## 6. Approvals

Erenumab was approved in the US for the preventive treatment of migraines in adults, based on positive Phase II and III results. It has also received a positive opinion in the EU for the prophylaxis of migraines in adults who have at least four migraine days/month. Erenumab was the first anti-CGRP/R mAb that was globally approved by both FDA and EMA for migraine prevention in 2018 [90]. The FDA approved galcanezumab as a once-monthly subcutaneous injection for the preventive treatment of migraines in adults in September 2018. The EMA also issued, for the prophylaxis of migraine in adults who have at least four migraine days/month, a positive opinion regarding the use of galcanezumab [91]. The agent has been tested in cluster headache prevention in two trials and it is approved for cluster headache prevention by the FDA but not by the EMA [92]. In September 2018, fremanezumab was also approved by the US FDA for the preventive treatment of migraines in adults and later on by the EMA [93]. In February 2020, eptinezumab, the first IV administered anti-CGRP/R mAb, was approved in the USA for the preventive treatment of migraine in adults and is under review by the EMA [79].

## 7. Guidelines for the Use of mAbs in Migraine (American Headache Society/European Headache Federation)

The American Headache Society (AHS), published, in 2018, a consensus position statement on integrating new migraine treatment into clinical practice [94]. It provides guidelines and indications for initiating treatment with anti-CGRP/R mAbs to achieve cost-effective care. Specifically, anti-CGRP/R mAbs initiation is recommended in patients with debilitating low-frequency EM, high-frequency EM or CM and intolerance or inadequate response to a 6-week trial of at least two preventive medications (e.g., topiramate, valproate, beta-blockers, tricyclic antidepressants, serotonin and noradrenaline r inhibitors (SNRIs)). Another recommendation in CM patients is inadequate response to a minimum of two quarterly injections (6 months) of onabotulinumtoxinA or inability to tolerate the treatment. AHS recommends assessment of the benefits of anti-CGRP/R mAbs after 3 months of treatment for those administered monthly and 6 months for those with quarterly administration of the treatment and the headache specialists may continue their administration only if treatment benefits are documented.

In 2019, the European Headache Federation (EHF) published guidelines on the use of monoclonal antibodies acting on the calcitonin gene-related peptide or its receptor for migraine prevention [95]. EHF found low-/high-quality evidence to recommend eptinezumab, erenumab, fremanezumab, and galcanezumab in patients with EM and medium-/high-quality evidence to recommend erenumab, fremanezumab and galcanezumab in CM patients. EHF (Expert Opinion Statement) recommends initiation of available anti-CGRP/R mAbs in patients with EM or CM who have failed at least two of the available medical treatments or who cannot use other preventive treatments due to side effects, poor compliance or comorbidities. EHF recommends a longer monitoring period than AHS and treatment discontinuation if there are not specific benefits recorded after 6–12 months. It is also stated that, in patients with CM and MOH, the use of anti-CGRP/R mAbs can be initiated before or after withdrawal of acute medications. Several national headache societies have published recommendations on the use of anti-CGRP/R mAbs for migraines [96,97,98].

## 8. Conclusions

There is no doubt that CGRP modulates cephalic pain neurotransmission. It is a potent vasodilator with excessive nociceptive properties. However, theoretical qualms concern the intensity of CGRP’s involvement in cephalic pain, i.e., how much this peptide controls migraine-related neurotransmission and how independent it is of other neurotransmission systems. Safety data from clinical trials and real-world evidence in migraine prevention verify the specificity of CGRP in the pathophysiology of migraines, i.e., that it plays an important role in the neurotransmission of pain more than other physiological actions, such as regulating blood pressure, for example. On the other hand, efficacy data from clinical trials and real-world evidence in migraine prevention indicate a complexity in migraine-related neurotransmission and that CGRP is not the only system that mediates migraine-related signals. In other words, the migraine-like sensitivity of CGRP is rather limited. Notably, CGRP targeting therapies almost eliminate migraines in a percentage of patients whose clinical phenotypes and characteristics have not yet been fully identified, indicating that CGRP’s sensitivity to migraine development depends on additional factors, most probably genetic ones. From a clinical point of view, further investigation is needed to verify the long-term safety and the consistency of efficacy of the anti-CGRP mAbs in migraine prevention. It is important to mention that not only controlled clinical studies but also real-world evidence is needed to evaluate the efficacy and AEs of anti-CGRP/R mAbs. From the RWE point of view, the new treatments go under the “crash test”, administered in patients with comorbidities and those highly refractory to treatments. Furthermore, real-world data from a cohort study including adolescents with refractory headache disorders revealed a treatment benefit [99]. This is encouraging as pediatric patients do not have many therapeutic options in their arsenal so far. Other aspects requiring more research include co-administration of anti-CGRP mAbs with other prophylactic anti-migraine agents and their effect on specific comorbidities such as depression and anxiety, as preliminary evidence suggests. Only in one monoclonal antibody—targeting the CGRP receptor—have additional AEs (constipation and rare deregulation of arterial hypertension) been recorded in real-world surveys. Is this related to the selective effect on the receptor, or is it a random observation, possibly related to the longer clinical use of this agent? Moreover, matters that arise concerning the difference in efficacy and effectiveness of anti-CGRP/R mAbs could be explained by genetic differences and pharmacogenetic research may unravel the pathogenetic mechanism. These queries along with potential risk for cerebrovascular AEs will be answered in the near future. Until then, close monitoring and implementation of the guidelines recommended by international and national headache societies is required to improve migraineurs quality of life, which remains the final goal of any medical interpretation.

## Data Availability

Data sharing not applicable.
